# Cerebrovascular Involvement in Transthyretin Amyloid Cardiomyopathy

**DOI:** 10.3390/jcm13154474

**Published:** 2024-07-31

**Authors:** Lukas Haider, Lore Schrutka, Emanuele Tommasino, Nicolas Avanzini, Sven Hauck, Nikolaus Nowak, Christian Hengstenberg, Diana Bonderman, Majda Thurnher

**Affiliations:** 1 Department of Biomedical Imaging and Image-Guided Therapy, Medical University of Vienna, 1090 Vienna, Austria; lukas.haider@meduniwien.ac.at (L.H.); emanuele.tommasino@gmail.com (E.T.); sven.hauck@meduniwien.ac.at (S.H.); nikolaus.nowak@outlook.com (N.N.); majda.thurnher@meduniwien.ac.at (M.T.); 2 Department of Internal Medicine II, Division of Cardiology, Medical University of Vienna, 1090 Vienna, Austria; lore.schrutka@meduniwien.ac.at (L.S.); nicolas.avanzini@hotmail.com (N.A.); christian.hengstenberg@meduniwien.ac.at (C.H.); 3 Department of Cardiology, Clinic Favoriten, 1100 Vienna, Austria

**Keywords:** anticoagulation, cardiac amyloidosis, cerebral small vessel disease, ischemia, silent stroke

## Abstract

**Background**: Intracardiac thrombosis is common in transthyretin amyloid cardiomyopathy (ATTR-CM), and patients are at risk for thromboembolic events. However, silent cerebral infarcts and the extent of cerebral small vessel disease in patients with cardiac amyloidosis are unknown. **Methods**: Thirty-two consecutively selected ATTR-CM patients were prospectively studied by cerebral magnetic resonance imaging (cMRI) and compared with 43 CHA_2_DS_2_-VASc-matched controls (Co). Structural clinical standard cMRI sequences and features of cerebral vessel involvement were included and quantified by two board certified neuroradiologists in consensus blinded to clinical status. Group differences were estimated using generalized (logistic) linear regression models adjusting for vascular risk factors based on the CHA_2_DS_2_-VASc score. **Results**: The median CHA_2_DS_2_-VASc score was 4 for ATTR-CM and Co (*p* = 0.905). There were no differences between groups in the frequency of current or former smokers (*p* = 0.755), body-mass-index > 30 (*p* = 0.106), and hyperlipidemia (*p* = 0.869). The number of territorial infarcts (4 vs. 0, *p* = 0.018) was higher in ATTR-CM compared to Co, as was the mean number of cerebral microbleeds (1.4 vs. 0.3, *p* ≤ 0.001) and the number of Virchow–Robin spaces (43.8 vs. 20.6, *p* ≤ 0.001). Lacunar lesion presence was higher in ATTR-CM (6 vs. 2, *p* = 0.054). CHA_2_DS_2_-VASc score, atrial fibrillation, anticoagulation, and the interaction term of CHA_2_DS_2_-VASc score and atrial fibrillation did not affect the probability of a territorial ischemic lesion or lacunar lesion in logistic regression modeling. **Conclusions**: In patients with ATTR-CM free from clinically apparent neurological symptoms, cMRI revealed unreported significant small cerebral vessel disease and territorial ischemia. Our findings may support low thresholds for anticoagulation and cMRI in patients with ATTR-CM.

## 1. Introduction

Transthyretin amyloid cardiomyopathy (ATTR-CM) is a life-threatening disease that arises from the accumulation of insoluble fibrous deposits in the heart [1]. Accumulation of misfolded monomers or oligomers in the myocardium causes progressive organ dysfunction, which manifests as heart failure with restrictive physiology [2]. Although the prevalence of ATTR-CM is uncertain and probably underestimated, studies have reported a prevalence of 13% in patients with heart failure with preserved ejection fraction [3], 16% in patients undergoing transcatheter aortic valve replacement for severe aortic stenosis [4] and 5% in patients with presumed hypertrophic cardiomyopathy [5]. Therefore, ATTR-CM is increasingly being recognized by the cardiology community, especially since the diagnosis can be made non-invasively with nuclear scintigraphy in the absence of monoclonal protein [6] and pharmaceutical therapy is now available that slows or halts ATTR-CM progression and favorably affects clinical outcomes [7,8,9].

The clinical course of CA is primarily characterized by heart failure. However, amyloid infiltration causes a wide spectrum of myocardial dysfunction that can lead to various other cardiac manifestations [10]. Infiltration of the atrial walls, severe atrial dilatation, impaired atrial strain, and increased ventricular filling pressure due to the restrictive physiology typical of advanced forms of cardiac amyloidosis can lead to rhythm disturbances with a high prevalence of atrial fibrillation [11]. Intracardiac thrombus formation is found in up to 18% of ATTR-CM patients and may occur even in sinus rhythm and in those receiving adequate anticoagulant therapy [12,13,14]. This increased risk was attributed to systolic and diastolic ventricular dysfunction and loss of atrial mechanical function, best measured by speckle tracking echocardiography [15,16], although higher rates of thrombi in light-chain (AL) amyloidosis patients with younger age and less atrial fibrillation might suggest other mechanisms [17]. Consequently, the rate of thromboembolic events is high and occurs despite antithrombotic treatment or in sinus rhythm [18,19]. Silent brain infarctions, also known as covert strokes, are ischemic events that are not clinically apparent. These infarcts, as well as cerebral small vessel disease, can be identified by brain imaging and have been associated with cognitive decline, dementia, increased risk of stroke, and increased mortality in population-based studies [20,21,22,23,24]. The incidence of silent cerebral infarcts and cerebral involvement in ATTR-CM however has not been studied. Given the high risk of thromboembolism, the aim of this study was to prospectively perform cerebral magnetic resonance imaging (cMRI) in consecutive individuals diagnosed with ATTR-CM.

## 2. Material and Methods

### 2.1. Subjects and Study Design

Consecutive patients were enrolled in the ATTR-CM observational registry at the Department of Cardiology of the Medical University of Vienna and were referred to cMRI after definite diagnosis of ATTR-CM. Patients with known pre-existing brain diseases such as (hemorrhagic-) infarction, intracranial hemorrhage, metastases, primary brain tumor, infectious or inflammatory disease, traumatic brain injury were excluded, as well as patients that previously underwent ablation of atrial fibrillation. Other reasons for exclusion were contraindications for cMR examination (including non-MR compatible pacemaker) and claustrophobia. The study was approved by the local ethics committee (EK #796/2010) and conducted according to good clinical practice as outlined in the declaration of Helsinki. Written informed consent was collected from all patients before enrollment in the institutional registry. Patients or the public were not involved in the design, or conduct, or reporting, or dissemination plans of our research

### 2.2. Clinical Definitions

Based on the non-invasive diagnostic algorithm by Gillmore and colleagues in June 2016 [6], ATTR-CM was diagnosed in patients with significant myocardial tracer uptake (Perugini grade ≥ 2) on bone scintigraphy and absence of paraprotein detected by serum immunofixation, urine immunofixation, and serum assay for free light chains. After 2016, the collection of endomyocardial biopsies for the diagnosis of ATTR became necessary only when non-invasive test results were ambiguous or unclear. When cardiac ATTR was diagnosed, patients were offered sequencing of the TTR gene to detect the presence of hereditary ATTR-CM (hATTR-CM), otherwise a wild-type form (wtATTR-CM) was assumed [25]. 

Atrial fibrillation was ascertained based on at least one in-hospital and/or ambulatory diagnosis of atrial fibrillation. Clinical characteristics, current treatment regimens, and anticoagulation therapy were recorded. The presence of comorbidities was recorded according to the respective guidelines [26,27]. The CHA_2_DS_2_-VASc (Congestive heart failure, Hypertension, Age ≥ 75 years, Diabetes mellitus, prior Stroke or TIA or thromboembolism, Vascular disease (e.g., peripheral artery disease, myocardial infarction, aortic plaque), Age 65–74 years and Sex category (i.e., female sex)) score for stroke risk in AF [28] was calculated for each patient. 

### 2.3. cMRI Acquisition

cMRI was performed over a recruitment period of 27 months, on a 3T Siemens Vida, including structural clinical standard cMRI sequences: 3D FLAIR (Repetition Time: 8500 ms, Echo Time: 87 ms, Inversion Time: 2440 ms, resolution: 1 mm isotropic), 3D T1 MPRAGE (Repetition Time: 2000 ms, Echo Time: 3.26 ms, Inversion Time: 1010 ms, resolution: 0.7 mm isotropic), 3D T1 black blood (Repetition Time: 700 ms, Echo Time: 11 ms, Flip Angle: 120°, resolution: 0.9 mm isotropic), coronally acquired T2-TSE (Repetition Time: 5130 ms, Echo Time: 89 ms, resolution: 0.5 × 0.5 × 2 mm), axially acquired DWI (b0 and b100, Repetition Time: 3130 ms, Echo Time: 86.2 ms, resolution: 1.5 × 1.5 × 5 mm), and SWI (Repetition Time: 28 ms, Echo Time: 20 ms, Flip Angle: 15°, resolution: 1.5 × 1.5 × 2 mm).

### 2.4. cMRI Analysis

Features of cerebral large vessel involvement, including territorial ischemia, dolichoectasia and vessel wall imaging, as well as cerebral microvascular involvement, including counts of enlarged perivascular spaces, lacunar lesions and microbleeds, by previously defined criteria [29], were quantified by two board certified neuroradiologists in consensus blinded to clinical status.

### 2.5. Statistical Analysis

Linear data are provided with the mean (standard deviation), and ordinal data with the median (total range). Group differences in cMRI measures were estimated using generalized (logistic) linear regression models adjusting for vascular risk factors based on the CHA_2_DS_2_-VASc score as applicable. Group differences in clinical data were estimated with the Chi-square test, ANOVA and Wilcoxon rank sum test depending on the data type and normal distribution, which was assessed with the Kolmogorov–Smirnov normality test. The statistics analysis was computed with RStudio 2024.04.2+764.

## 3. Results

### 3.1. Clinical Characteristics

A total of 75 patients underwent cMRI. A total of 32 patients with definite ATTR-CM, including 26 (81.2%) with wtATTR and 6 (18.8%) with hATTR amyloidosis. In total, 43 CHA_2_DS_2_-VASc-matched patients with psychiatric indications and cephalea served as controls (Co). Detailed baseline characteristics of ATTR-CM and Co are presented in Table 1. Median CHA_2_DS_2_-VASc score values (ATTR-CM: 4 (range: 1–7), Co: 4 (range: 0–7)) did not differ between ATTR-CM and Co (*p* = 0.905); in detail, congestive heart failure (ATTR-CM: 12 (37.5%), Co: 15 (34.9%), *p* = 0.815), hypertension (ATTR-CM: 24 (75.0%), Co: 31 (72.1%), *p* = 0.778), age (ATTR-CM: 74.7 (SD 8.0), Co: 77.1 (SD 9.1), *p* = 0.239), diabetes, all of which type 2 (ATTR-CM: 12 (37.5%), Co: 13 (30.2%), *p* = 0.509), no previous strokes or transient ischemic attacks, presence of peripheral vascular disease, e.g., peripheral artery disease, myocardial infarction or aortic plaques (ATTR-CM: 21 (65.6%), Co: 21 (48.8%), *p* = 0.147), and sex (CA: female: 7 (21.9%), Co: 13 (25.3%), *p* = 0.418). 

Furthermore, we did not observe statistically significant differences in the frequency of current or former smokers (ATTR-CM: 3 (9.4%), Co: 5 (11.6%), *p* = 0.755), individuals with a body-mass-index (BMI) > 30 (ATTR-CM: 5 (15.6%), Co: 2 (4.7%), *p* = 0.106) and hyperlipidemia (ATTR-CM: 11 (34.4%), Co: 14 (32.6%), *p* = 0.869), between individuals with ATTR-CM and Co. Atrial fibrillation was diagnosed more often in ATTR-CM (ATTR-CM: 16 (44.5%), Co: 11 (25.6%), *p* = 0.029), and ATTR-CM patients were more frequently anticoagulated (ATTR-CM: 18 (50.0%), Co: 10 (23.3%), *p* = 0.004) than controls. In both groups, patients on anticoagulation were treated more commonly with direct oral anticoagulants (DOAC) than with vitamin K antagonists (VKA). Of the 18 ATTR-CM patients on oral anticoagulation, two were treated with warfarin and 16 with DOACs. Among those on DOACs two patients received rivaroxaban, one dabigatran, seven apixaban and six edoxaban. In the controls, four were treated with apixaban, four with edoxaban, one with rivaroxaban and one with warfarin. (Table 1). None of the patients in our cohort underwent left atrial appendage occlusion.

### 3.2. Cerebrovascular MRI Findings

**Figure 1:**
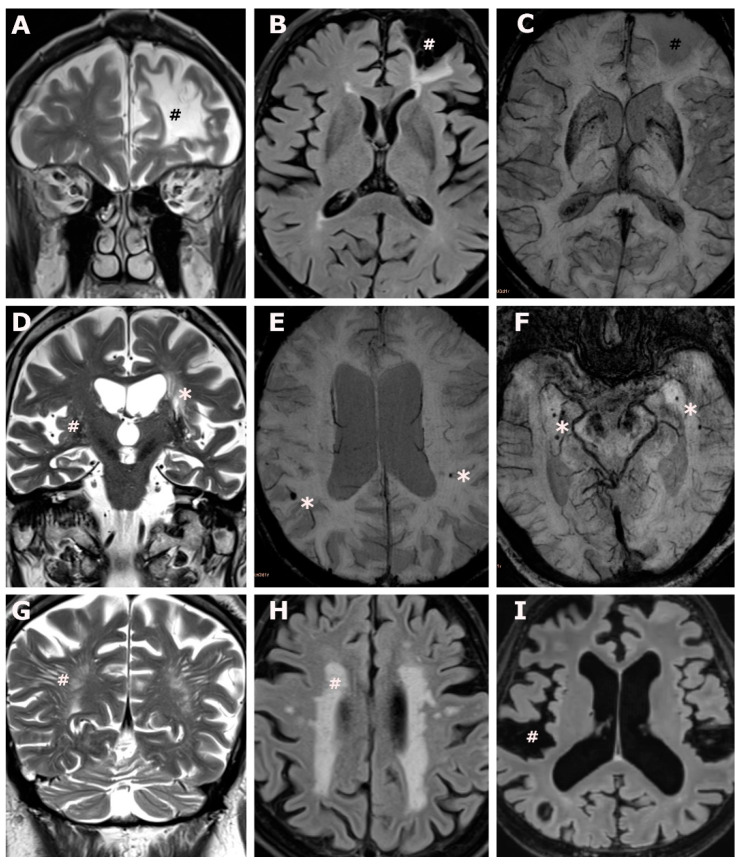
Brain involvement in transthyretin amyloid cardiomyopathy. (**A**–**C**): An 83-year-old male ATTR-CM subject, CHA_2_DS_2_-VASc score 5, with atrial fibrillation under anticoagulation, and no report of ischemic brain lesions at the time of brain MR acquisition. Coronal T2 weighted images (**A**) and axial fluid attenuated inversion recovery weighted images (**B**) show a non-recent, cystic-gliotic, lesion in the territory of the anterior cerebral artery (#) extending to the watershed area, no evidence of hemorrhagic transformation on axial susceptibility weighted images (**C**), overall consistent with an old ischemic stroke. (**D**): An 88-year-old male ATTR-CM subject, CHA_2_DS_2_-VASc score 5, atrial fibrillation under anticoagulation, and no report of ischemic brain lesions at the time of brain MR acquisition. Coronal T2 weighted images (**D**) show lacunar lesions (*) and enlarged perivascular spaces in the deep grey matter. (**E**,**F**): An 88-year-old male ATTR-CM subject, CHA_2_DS_2_-VASc score 4, no atrial fibrillation, no anticoagulation, and no report of brain involvement at the time of brain MR acquisition (**E**) and a 76-year-old male ATTR-CM subject, CHA_2_DS_2_ VASc score 6, no atrial fibrillation, no anticoagulation, and no report of brain involvement at the time of brain MR acquisition (**F**). Axial susceptibility weighted images (**E**,**F**) show peripherally located, bilateral microbleeds (*). (**G**): A 68-year-old male ATTR-CM subject, CHA_2_DS_2_-VASc score 3, with atrial fibrillation under anticoagulation, and no report of brain involvement at the time of brain MR acquisition. Coronal T2 weighted images (**G**) show extensive largening of perivascular spaces in the occipital deep white matter (#). (**H**): A 79-year-old female ATTR-CM subject, CHA_2_DS_2_-VASc score 3, no atrial fibrillation, no anticoagulation, and no report of brain involvement at the time of brain MR acquisition. Axial fluid attenuated inversion recovery weighted images (**H**) show confluent white matter hyperintensities in the deep- and periventricular white matter (#), consistent with Fazekas grade 3. (**I**): Same subject as (**E**), axial fluid attenuated inversion recovery images show extensive global widening of the subarachnoid space (#) and ventricular enlargement, consistent with global brain atrophy.

Group differences in the distribution of cMRI measures between ATTR-CM and Co were estimated with linear regression models, controlling for vascular risk factors based on the CHA_2_DS_2_-VASc score (Table 2). Representative imaging findings are provided in Figure 1. We detected four cases with territorial infarctions, all of which were present in ATTR-CM (ATTR-CM: n = 4 (12.5%) vs. Co: n = 0 (0.0%), *p* = 0.018). While lacunar lesions were detected more frequently in individuals with ATTR-CM (ATTR-CM: n = 6 (18.8%) vs. Co: n = 2 (4.7%)), the group difference did not reach the level of statistical significance (*p* = 0.054), Table 2.

Overall cerebral microbleeds were more frequently observed in ATTR-CM than in Co (CA: mean 1.4 (SD 1.4) vs. Co: mean 0.3 (SD 0.7), *p* ≤ 0.001), as were cerebral microbleeds in a lobar distribution (CA: mean 1.3 (SD 1.4) vs. Co: mean 0.2 (SD 0.6), *p* ≤ 0.001), whereas cerebral microbleed in the deep grey matter were found in comparable distribution in both groups (ATTR-CM: mean 0.1 (SD 0.3) vs. Co: mean 0.0 (SD 0.2), *p* = 0.379). 

The mean Virchow–Robin space counts were significantly higher in ATTR-CM than in Co, (CA: 43.8 (SD 18.4) vs. Co: 20.6 (SD 9.0), *p* ≤ 0.001).

We did not observe significant group differences for dolichoectatic expansion of the basal cerebral arteries (CA: 20.6 (18.5%) vs. Co: 14.4 (26.5%), *p* = 0.271) and vessel wall hemorrhage (CA: 7 (21.9%) vs. Co: 6 (14.0%), *p* = 0.367).

### 3.3. Territorial Ischemia

Of the four subjects with territorial ischemia, all of which were non-recent (i.e., cystic gliotic), microbleeds were found in all of them, three subjects also had additional lacunar lesions, and one subject had dolichoectatic elongation of the basal arteries. All four subjects with territorial infarct had wtATTR amyloidosis. Vascular risk factors measured by the CHA_2_DS_2_-VASc score were present in three of the four subjects and atrial fibrillation was previously diagnosed in one of the four subjects. Two of the four patients were treated with anticoagulation. CHA_2_DS_2_-VASc score, atrial fibrillation, anticoagulation as well as the interaction term of CHA_2_DS_2_-VASc score and atrial fibrillation had no effect on the probability of having a territorial ischemic lesion or lacunar lesions using logistic regression modeling (Table 3). Similarly, a sub-analysis of the type of anticoagulation did not reach significant effect sizes on territorial ischemia (DOAK: OR: 0.19, CI: 0.01–3.06, *p* = 0.208 and VKA: OR: 0.0, CI: NA, *p* = 0.995) and lacunar infarct occurrences (DOAK: OR: 1.02, CI: 0.12–9.89, *p* = 0.989 and VKA: OR: 0.0, CI: NA, *p* = 0.995). 

A further sub-analysis of our cohort that included left ventricular ejection fraction (LVEF) and left atrial volume (LAV) from transthoracic echocardiograms, showed no significant effect on the probability of having silent ischemia (LVEF: OR: 1.01, CI: 0.90–1.15, *p* = 0.830 and LAV: OR: 1.03, CI: 0.96–1.12, *p*= 0.433) or lacunar infarction (LVEF: OR: 0.99, CI: 0.90–1.09, *p*= 0.864 and LAV: OR: 0.98, CI: 0.92–1.02, *p* = 0.393), respectively.

## 4. Discussion

This cMR-based observational study provides an estimate of cerebral micro- and macrovascular involvement in patients with ATTR-CM exceeding that of age and risk factor matched controls. Our results suggest that the high rate of territorial infarcts and cerebral small vessel disease in neurologically asymptomatic ATTR-CM patients is not limited to individuals with atrial fibrillation and that anticoagulation therapy does not seem to fully protect against these serious complications.

Risk prediction models such as the CHA_2_DS_2_-VASc score have been introduced to predict the risk of cerebral vascular events in patients with atrial fibrillation [28]. Atrial fibrillation is present in approximately 40% of patients with ATTR-CM and was found to be associated with a significant risk of stroke and systemic thromboembolism regardless of the CHA_2_DS_2_-VASc score [30]. In this regard, oral anticoagulation appears to be a safe preventive measure against ischemic events and is preferred once atrial fibrillation is detected in ATTR-CM [31,32]. However, ATTR-CM patients are likely to be at particularly increased risk for intracardiac thrombus formation, which has already been observed in patients in sinus rhythm and with adequate anticoagulation [12,13,14]. In patients with coexisting atrial fibrillation and amyloidosis who underwent left atrial appendage occlusion, maintaining half the DOAC dose proved to be the safest stroke prevention strategy, indicating that the risk of thrombus formation remains high even after left atrial appendage occlusion [33]. In this study, we observed a strikingly high number of cerebral infarctions, with most patients already treated with oral anticoagulation. Although vascular risk factors were present in almost all patients, the CHA_2_DS_2_-VASc score did not seem to influence the likelihood of the occurrence of ischemic lesions. Moreover, these changes did not appear to be dependent on the presence of atrial fibrillation. Our observation is consistent with another observational study examining arterial embolism in ATTR-CM, in which a significant proportion of events occurred even despite anticoagulation therapy or in patients in sinus rhythm [18].

While cerebral amyloid angiopathy, considered a subtype of cerebral small vessel disease, is characterized by the ß-amyloid accumulation in the walls of small cortical and leptomeningeal arterioles and arteries, there have been no reports of cerebral or cerebrovascular amyloid deposition in ATTR-CM. Cerebral small vessel disease has been associated with cognitive decline, dementia, increased risk of stroke, and increased mortality in population-based studies [20,21,22,23,24]. The main imaging features of cerebral small vessel disease are white matter lesions, enlarged perivascular spaces and microbleeds.

Microbleeds, defined as hypointense foci visible on susceptible-weighted cMRI sequences are found in up to one-third of patients with ischemic stroke [34]. Because of their hemorrhagic histopathological substrate, they have been historically associated with a risk of intracerebral hemorrhage. This has led to concerns about the safety of anticoagulation use in patients with cerebral microbleeds. However, there is increasing evidence that cerebral microbleeds are not only markers of hemorrhage but even more so of future ischemic events [35] and the use of oral anticoagulants in the presence of microbleeds has not shown any impact on clinical outcomes to date [36]. Besides silent territorial infarcts, we found a high prevalence of microbleeds. The predominantly lobar distribution pattern of microbleeds that we observed in ATTR-CM patients has also been previously noted in cerebral ß-amyloid angiopathy, which may suggest a similar pathomechanism [37].

Enlarged perivascular spaces (also known as Virchow–Robin spaces), refer to the space surrounding blood vessels, as they penetrate the brain parenchyma. Increased counts and volume of perivascular spaces have been reported in cerebral small vessel disease [38]. Enlarged perivascular spaces are believed to reflect underlying changes in the brain’s vasculature, the blood brain barrier and brain atrophy [29]. Their frequency has been correlated with cognitive impairment [39], and lacunar infarction [38]. However, in a metanalysis, not differentiating between lacunar and non-lacunar infarction, no association between enlarged perivascular space and stroke risk was found [40]. Although the significance of Virchow–Robin spaces has not been fully elucidated, the increased number of counts in patients with ATTR-CM compared with controls suggests the presence of substantial microvascular changes.

Lacunar infarcts are small, localized areas of infarction that result from the occlusion or blockage of small penetrating arteries within the brain [29]. Although lacunar strokes may occasionally result from mechanisms of brain ischemia, such as cardiac embolism or carotid artery stenosis, most result from intrinsic diseases of the small deep perforating arteries [41]. We also found a trend toward an increased number of lacunar infarcts, but this did not reach statistical significance. 

In view of our cMR tomographic findings indicating a high rate of silent embolic infarcts, our results may further support low thresholds for anticoagulation therapy and brain imaging in patients with ATTR-CM. Although the exact cause of the large number of chronic small vessel changes in ATTR-CM patients remains unclear, efforts should be made to best control known risk factors such as hypertension, diabetes, and hypercholesterolemia to potentially prevent progression of the changes. 

### Limitations

While recruitment was prospective in the ATTR-CM cohort, the Co cohort was collected from a retrospective chart review of clinical data and while it well matched in terms of age, sex, and vascular risk factors, we cannot exclude bias towards brain pathology as clinical indications lead to the referral for a cMRI in these elderly subjects. However, indications were psychiatric and cephalea related in our Co cohort, and while these conditions are not associated with structural brain alterations per se and might have further decreased our sensitivity to detect group differences, it is unlikely that it introduced cases with less vascular pathology than would have been observed in healthy aging control cases.

Although the study controlled for vascular risk factors based on the CHA_2_DS_2_-VASc score, it is important to note that other unmeasured confounders such as lifestyle and comorbidities may still have influenced the results.

Patients presented without neurological complaints, but did not undergo standardized neurological examination, which could have provided probabilities of territorial ischemia prior to the examination and may have led to overlooking subclinical or minor neurological symptoms that could have provided additional context for the cMRI findings.

The relatively small sample size of 32 ATTR-CM patients limits the generalizability of the results and reduces the statistical power to detect small differences or rare outcomes. Therefore, this study should be regarded as exploratory, allowing for the recognition of significant changes despite the limited number of cases. However, these initial findings need to be further validated by larger studies

## 5. Conclusions

While brain involvement in ATTR-CM has not been systematically reported previously, we found substantial cerebral microvascular and macrovascular involvement in individuals without clinical neurologic symptoms who were prospectively recruited for cMRI. Our findings might further support low thresholds for anticoagulation therapy and brain imaging in patients with ATTR-CM.

## Figures and Tables

**Table 1:** Baseline characteristics of ATTR-CM patients and matched controls.

	Total (N = 75)	ATTR-CM (N = 32)	Controls (N = 43)	*p*-Value
Male sex, n (%)	55 (73.3%)	25 (78.1%)	30 (69.8%)	0.418 ^1^
Age at cMRI, mean years (SD)	76.1 (8.7)	74.7 (8.0)	77.1 (9.1)	0.239 ^2^
BMI >30 kg/m^2^, n (%)	7 (9.3%)	5 (15.6%)	2 (4.7%)	0.106 ^1^
Hyperlipidemia, n (%)	25 (33.3%)	11 (34.4%)	14 (32.6%)	0.869 ^1^
Diabetes mellitus type II, n (%)	25 (33.3%)	12 (37.5%)	13 (30.2%)	0.509 ^1^
Current or ex-smoker, n (%)	8 (10.7%)	3 (9.4%)	5 (11.6%)	0.755 ^1^
Hypertension, n (%)	55 (73.3%)	24 (75.0%)	31 (72.1%)	0.778 ^1^
Atrial fibrillation, n (%)	27 (36.0%)	16 (50.0%)	11 (25.6%)	0.029 ^1^
Congestive heart failure, n (%)	27 (36.0%)	12 (37.5%)	15 (34.9%)	0.815 ^1^
Vascular disease, n (%)	42 (56.0%)	21 (65.6%)	21 (48.8%)	0.147 ^1^
Oral anticoagulation, n (%)	28 (37.3%)	18 (56.2%)	10 (23.3%)	0.004 ^1^
Type of anticoagulation				0.103 ^1^
Apixaban, n (%)	11 (14.7%)	7 (21.9%)	4 (9.3%)	
Dabigatran, n (%)	1 (1.3%)	1 (3.1%)	0 (0.0%)	
Edoxaban, n (%)	10 (13.3%)	6 (18.8%)	4 (9.3%)	
Rivaroxaban, n (%)	3 (4.0%)	2 (6.2%)	1 (2.3%)	
VKA, n (%)	3 (4.0%)	2 (6.2%)	1 (2.3%)	
CHA_2_DS_2_-VASc-score, median points (IQR)	4 (0.0–7.0)	4 (1.0–7.0)	4 (0.0–7.0)	0.905 ^3^
Left ventricular ejection fraction, mean% (SD)	NA	53.8 (9.6)	NA	NA
Left atrial volume, mean mL (SD)	NA	79.6 (30.1)	NA	NA
Left ventricular global longitudinal strain, mean% (SD)	NA	−12.9 (3.8)	NA	NA

Legend: ATTR-CM, transthyretin amyloid cardiomyopathy; cMRI, cerebral magnetic resonance imaging; BMI, body mass index; VKA, vitamin K antagonist; DOAC, direct oral anticoagulant; CHA_2_DS_2_-VASc (Congestive heart failure, Hypertension, Age ≥ 75 years, Diabetes mellitus, prior Stroke, Vascular disease, Age 65–74 years, Sex category) score calculates stroke risk for patients with atrial fibrillation. Values are given as mean and standard deviation (SD), median and interquartile range (IQR), or total numbers (n) and percent (%); ^1.^ Pearson’s Chi-squared test; ^2.^ Linear Model ANOVA; and ^3.^ Wilcoxon rank sum test.

**Table 2:** Results from magnetic resonance imaging in ATTR-CM patients and controls.

	Total (N = 75)	ATTR-CM (N = 32)	Controls (N = 43)	*p*-Value
Territorial ischemic lesions, n (%)	4 (5.3%)	4 (12.5%)	0 (0.0%)	0.018 ^1^
Lacunar lesions, n (%)	8 (10.7%)	6 (18.8%)	2 (4.7%)	0.054 ^1^
Microbleeds, mean (SD)	0.7 (1.2)	1.4 (1.4)	0.3 (0.7)	≤0.001 ^1^
Centrally located microbleeds, mean (SD)	0.1 (0.3)	0.1 (0.3)	0.0 (0.2)	0.379 ^1^
Peripherally located microbleeds, mean (SD)	0.7 (1.2)	1.3 (1.4)	0.2 (0.6)	≤0.001 ^1^
Virchow–Robin spaces, mean (SD)	30.3 (17.8)	43.8 (18.4)	20.6 (9.0)	≤0.001 ^1^
Virchow–Robin spaces white matter, mean (SD)	15.2 (8.7)	22.6 (7.2)	9.9 (5.1)	≤0.001 ^1^
Virchow–Robin spaces deep grey matter, mean (SD)	10.5 (10.3)	15.8 (13.7)	6.7 (4.0)	0.001 ^1^
Virchow–Robin spaces hippocampus, mean (SD)	1.9 (1.3)	2.2 (1.1)	1.7 (1.3)	0.091 ^1^
Virchow–Robin spaces midbrain and infratentorial, mean (SD)	2.7 (2.2)	3.2 (2.4)	2.3 (1.9)	0.065 ^1^
Dolichoectatic elongation of basal arteries, n (%)	17.0 (23.5)	20.6 (18.5)	14.4 (26.5)	0.271 ^1^
T1 fat sat black blood vessel wall signal, n (%)	13 (17.3%)	7 (21.9%)	6 (14.0%)	0.367 ^1^

Legend: ATTR-CM, transthyretin amyloid cardiomyopathy. Values are given as mean and standard deviation (SD), median and interquartile range (IQR), or total numbers (n) and percent (%). Group differences in the distribution of cMRI measures between ATTR-CM and Co were estimated with linear regression models, controlling for vascular risk factors based on the CHA_2_DS_2_-VASc score. ^1^ Pearson’s Chi-squared test.

**Table 3:** Logistic regression model on the probability territorial stroke or lacunar infarct.

	Territorial Infarct (n = 4)			Lacunar Infarct (n = 6)		
Variable	Odds Ratios	95% Confidence Interval	*p*-Value	Odds Ratios	95% Confidence Interval	*p*-Value
Intercept	0.01	0.00–0.17	0.015	0.02	0.00–0.20	0.011
CHA_2_DS_2_-VASc score	1.46	0.77–3.65	0.308	1.58	0.91–3.38	0.150
Atrial fibrillation	1.13	0.00–1440.82	0.974	11.55	0.07–2224.00	0.326
Oral anticoagulation	4.92	0.28–68.84	0.234	0.79	0.08–7.25	0.836
CHA_2_DS_2_-VASc score: Atrial fibrillation	0.65	0.13–3.37	0.600	0.60	0.22–1.58	0.297

CHA_2_DS_2_-VASc (Congestive heart failure, Hypertension, Age ≥ 75 years, Diabetes mellitus, prior Stroke, Vascular disease, Age 65–74 years, Sex category) score calculates stroke risk for patients with atrial fibrillation.

## Data Availability

The raw data supporting the conclusions of this article may be made available by the authors upon reasonable request.
